# Supplementary material for: Characterization of the Gut and Skin Microbiome over Time in Young Children with IgE-Mediated Food Allergy
Source: Nutrients. 2024 Nov 19;16(22):3942. doi: 10.3390/nu16223942 (PMC11597333; doi:10.3390/nu16223942)
Supplement: Supplementary file 1 [file nutrients-16-03942-s001.zip › nutrients-3244778-supplementary.pdf]

nutrients MDPI

Supplementary Methods

Bacterial DNA isolation

Skin microbial DNA was extracted from the skin swab samples using a QIAmp DNA Microbiome Kit (Qiagen, Hildfeld, Germany) according to the manufacturer's instructions. Feces bacterial DNA was prepared from the feces samples using a Qiagen Power Soil Kit (Qiagen) according to the manufacturer's protocol. These isolated bacterial DNA samples were further processed for 16S rRNA gene library preparation.

Library preparation and sequencing for the 16S rRNA gene

Library preparation for the bacterial 16S rRNA gene was performed as described [41,73,74]. The hypervariable V1–V2 regions of the bacterial 16S rRNA gene were amplified by PCR using the 27 forward/338 reverse primer combination, employing a dual-index strategy. PCR was conducted in 25 µl volume, consisting of 1 µl template DNA, 4 µl of each forward and reverse primer, 0.25 µl Phusion Hot Start II DNA polymerase (0.5 units), 0.5 µl deoxynucleotide triphosphates (200 µM each), and 5 µl of hydrofluoric acid buffer. PCR condition was as follows: initial denaturation for 30 seconds at 98°C; 32 cycles of 9 seconds at 98°C, 30 seconds at 55°C, and 45 seconds at 72°C; and a final extension for 10 minutes at 72°C. The concentration of the PCR products was estimated on 1.5% agarose gels using the image software Quantum Capt, version 16.04 (Vilber Lourmat Deutschland, Eberhardzell, Germany), with a known concentration of DNA marker (100 bp DNA Ladder; Thermo Fisher Scientific, Dreieich, Germany) as the internal standard for band intensity measurement. The PCR products were pooled into approximately equimolar sub-pools, as indicated by band intensity. The sub-pool samples were purified using GeneJET NGS Cleanup Kit (Thermo Fisher Scientific). The concentration of purified sub-pools was quantified using qPCR using NEBNext Library Quant Kit for Illumina (New England BioLabs GmbH, Frankfurt am Main, Germany). The sub-pools were combined in one equimolar final pool for each library. The final pools were cleaned up with magnetic beads (MagSi-NGSPREP Plus; Steinbrenner Laborsysteme, Wiesenbach, Germany), and the Qubit dsDNA BR Assay Kit measured the concentration of the final library on a Qubit fluorometer (Thermo Fisher Scientific), and the size was determined by Bioanalyzer 2100 instrument using Agilent DNA 7500 Kit (Agilent Technologies, Waldbronn, Germany). The final library was sequenced on the Illumina MiSeq platform with version 2 (2 × 250 bp) chemistry (Illumina, Berlin, Germany).

Bioinformatics

Sequencing data from patients and controls (demultiplexed fastq files) were processed into operational taxonomic units (OTUs) using vsearch (v2.8.0) and UPARSE as implemented in USEARCH (v11.0.667) [75,76] as previously described [77]. In brief, forward and backward reads were merged (maxdiff = 2) and low-quality reads (maxee = 0.5) as well as chimeric sequences (reference RDP v9) were removed. Afterward, reads were clustered into OTUs, and taxonomy was assigned to each OTU using the SINTAX with GTDB r207 as a reference database [78,79]. OTUs not matching the kingdom Bacteria were removed to filter for potential contamination. Furthermore, OTUs matching the phyla Armatimonadota, Chloroflexota, Cyanobacteria, Dependentiae, Gemmatimonadota, Nitrospirota, Planctomycetota, CSP1-3, Eisenbacteria, Methylomirabilota, or Omnitrophota were removed as potential contaminants or wrongly assigned phyla. To further exclude potential contaminations, the frequency method of the decontam (v1.16.0) R package (threshold 0.2; 85 OTUs were removed) and the prevalence method (threshold 0.2; 26 OTUs removed) were applied [80]. Finally, OTUs occurring in less than 5 % of the samples were excluded, yielding 796 OTUs in 146 samples with an average of 72,717 contigs per sample (standard deviation ± 50,627).

Potential batch effects between patients and controls were assessed using MBECS (v1.0.0). The analysis showed no obvious batch effects between patients and controls [81].

Supplementary Figures

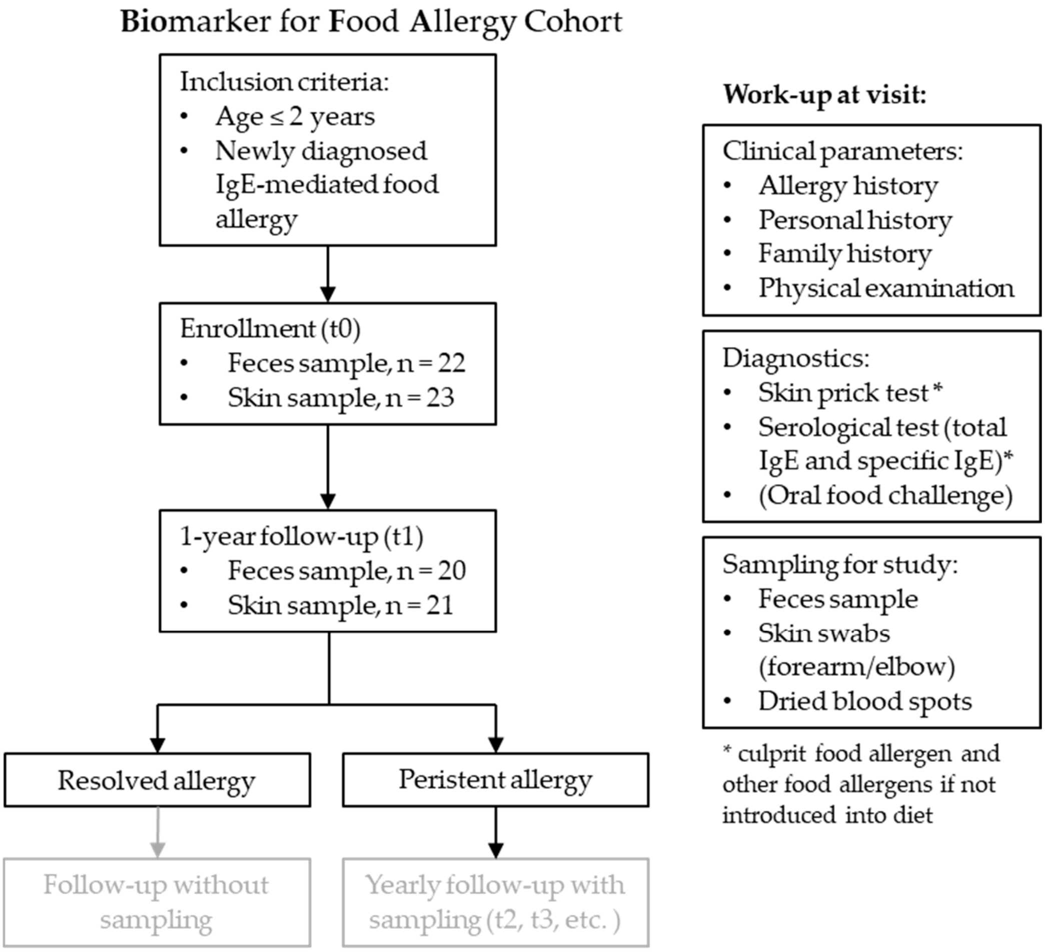

Supplementary Figure S1. Study design. Patients were a subset of the multicenter prospective BioFA (Biomarker for Food Allergy) cohort. Patients were enrolled at the age of 0-24 months when clinical parameters, diagnostics and sampling were performed. Follow-up was performed yearly at 6-18 months depending on the visit schedule. For this study, the microbiome of feces and skin samples from 23 patients was analyzed.

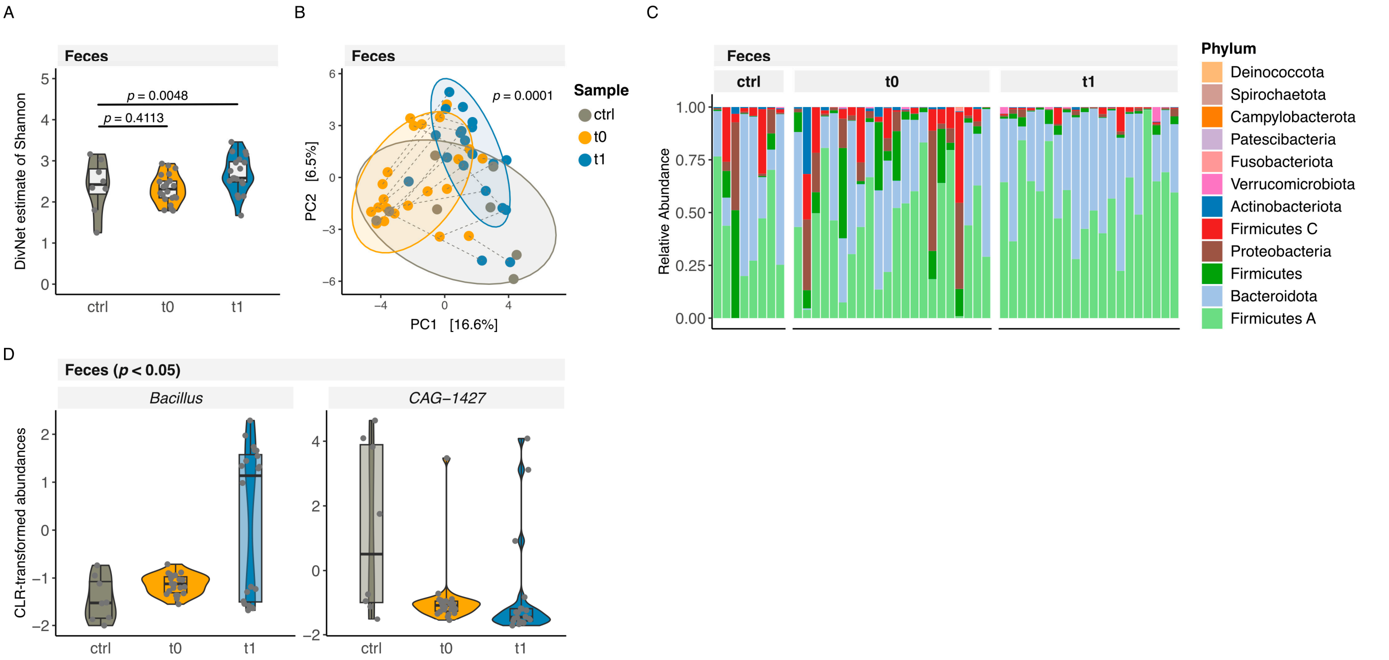

Supplementary Figure S2. Diversity and community composition of the gut of patients with IgE-mediated food allergy (FA) compared with controls. **(A)** Violin plot of alpha diversity (Shannon estimate; betta test) and **(B)** RDA (redundancy analysis) plot of community composition (beta diversity) of feces samples of controls and of patients at two different sampling time points (ctrl, sample of control; t0, sample of patients at enrollment; t1, sample of patient at 1-year follow-up; PC, principal coordinate; each dot represents one sample; grey dot, sample of control; yellow dot, sample of patient at enrollment (t0), blue dot, sample of patient at 1-year follow-up (t1); colored ellipses comprise 80% of the data). **(C)** Panel of relative abundances at phylum level of individual samples of feces of patients and controls. Violin plot of differences in clr-transformed genus abundances of feces **(D)**. (feces t0 n = 22, t 1 n = 20; significance level as indicated in figures).

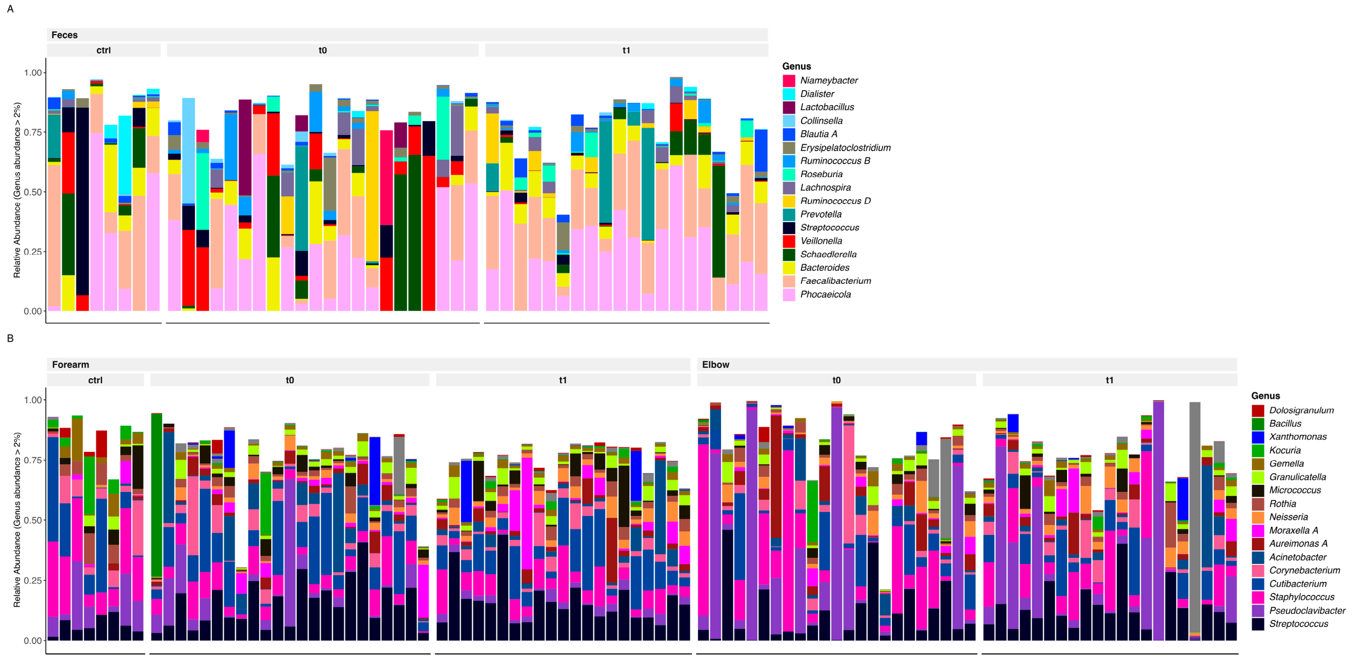

Supplementary Figure S3. Individual Taxonomic abundances at genus level. Relative abundances of the microbiome of feces **(A)** and skin **(B, forearm, elbow)** samples of patients with food allergy at different sampling timepoints and of controls (each column represents an individual sample; t0, sampling time point at enrollment; t1, sampling time point at 1-year follow up; feces t0 n = 22, t 1 n = 20; forearm/elbow t0 n = 23, t1 n = 21).

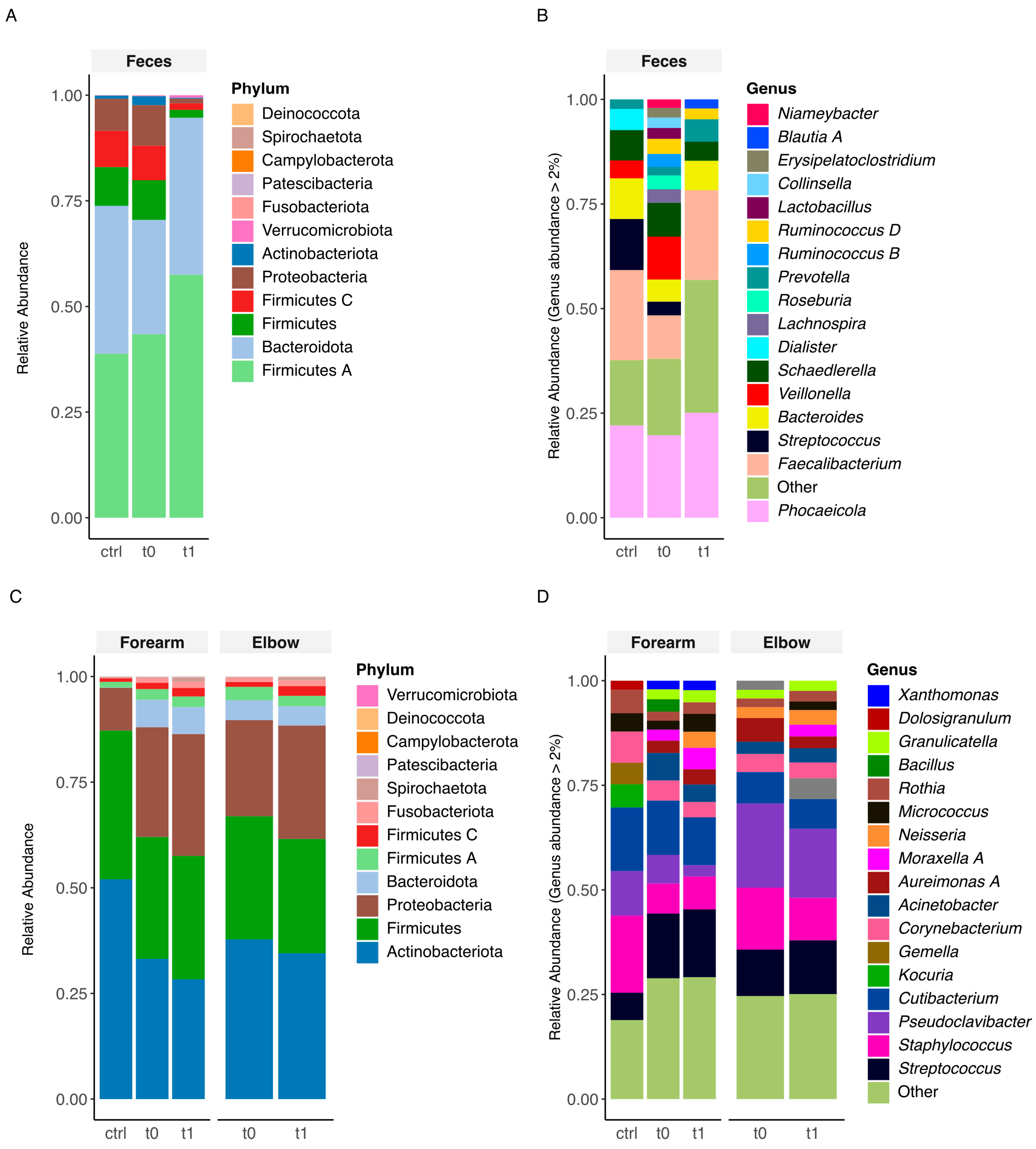

Supplementary Figure S4. Average taxonomic abundances of gut and skin microbiome of patients with IgE-mediated food allergy (FA) and controls. Relative abundances at phylum and genus level of the microbiome of feces **(A, B)** and skin **(C, D**; forearm, elbow) samples of patients at different sampling time points and of controls (each column represents the average relative abundance; t0, sampling time point at enrollment; t1, sampling time point at 1-year follow up).

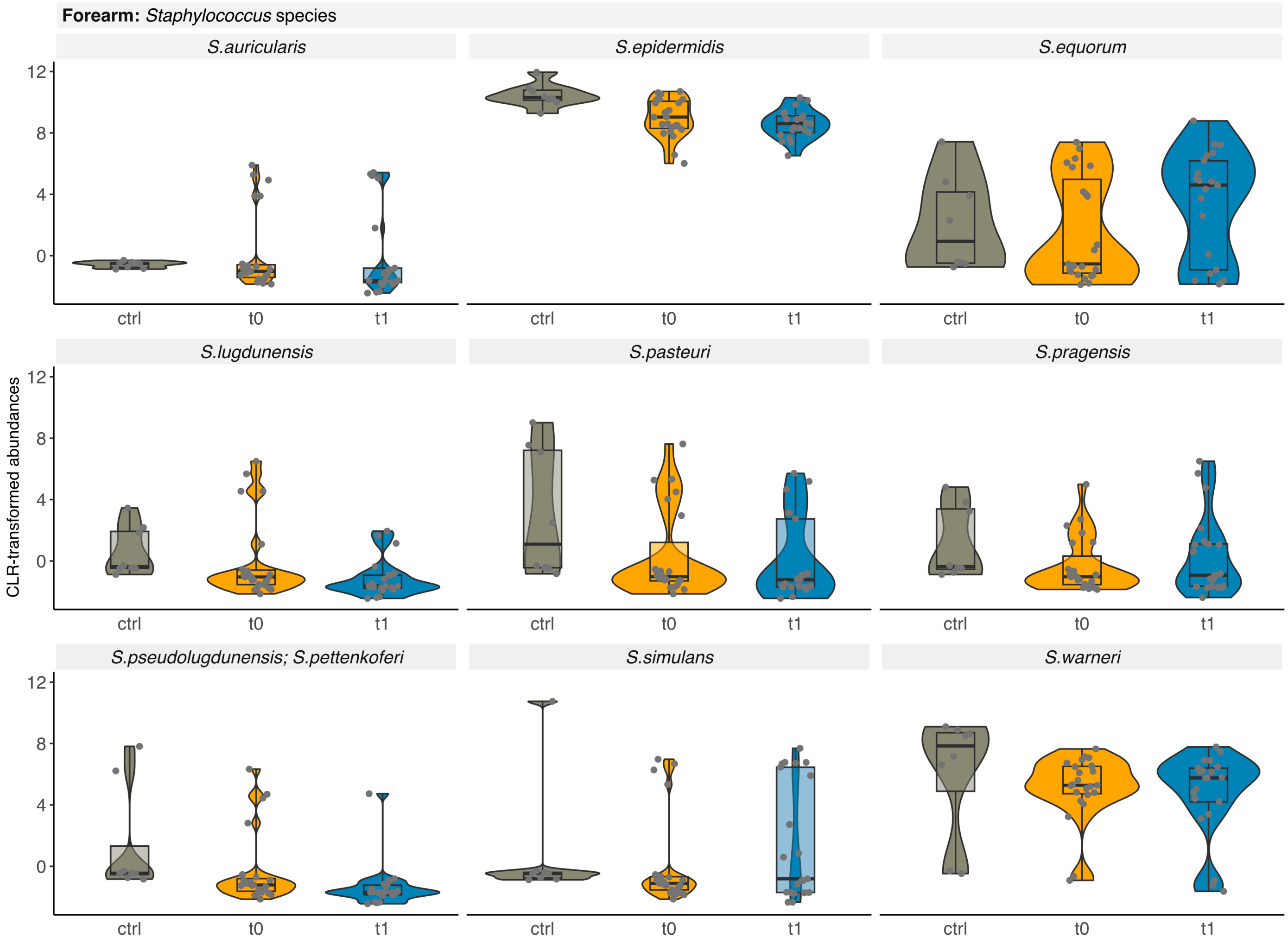

Supplementary Figure S5. Relative abundances of *Staphylococcus* species of patients with IgE-mediated food allergy (FA) and controls. Violin plots of clr- transformed abundances of *Staphylococcus* at species level of forearm samples of patients at different time points and controls. (ctrl, controls; t0, sampling at enrollment; t1, sampling at 1-year follow-up; forearm t0 n = 23, t0 n = 21).

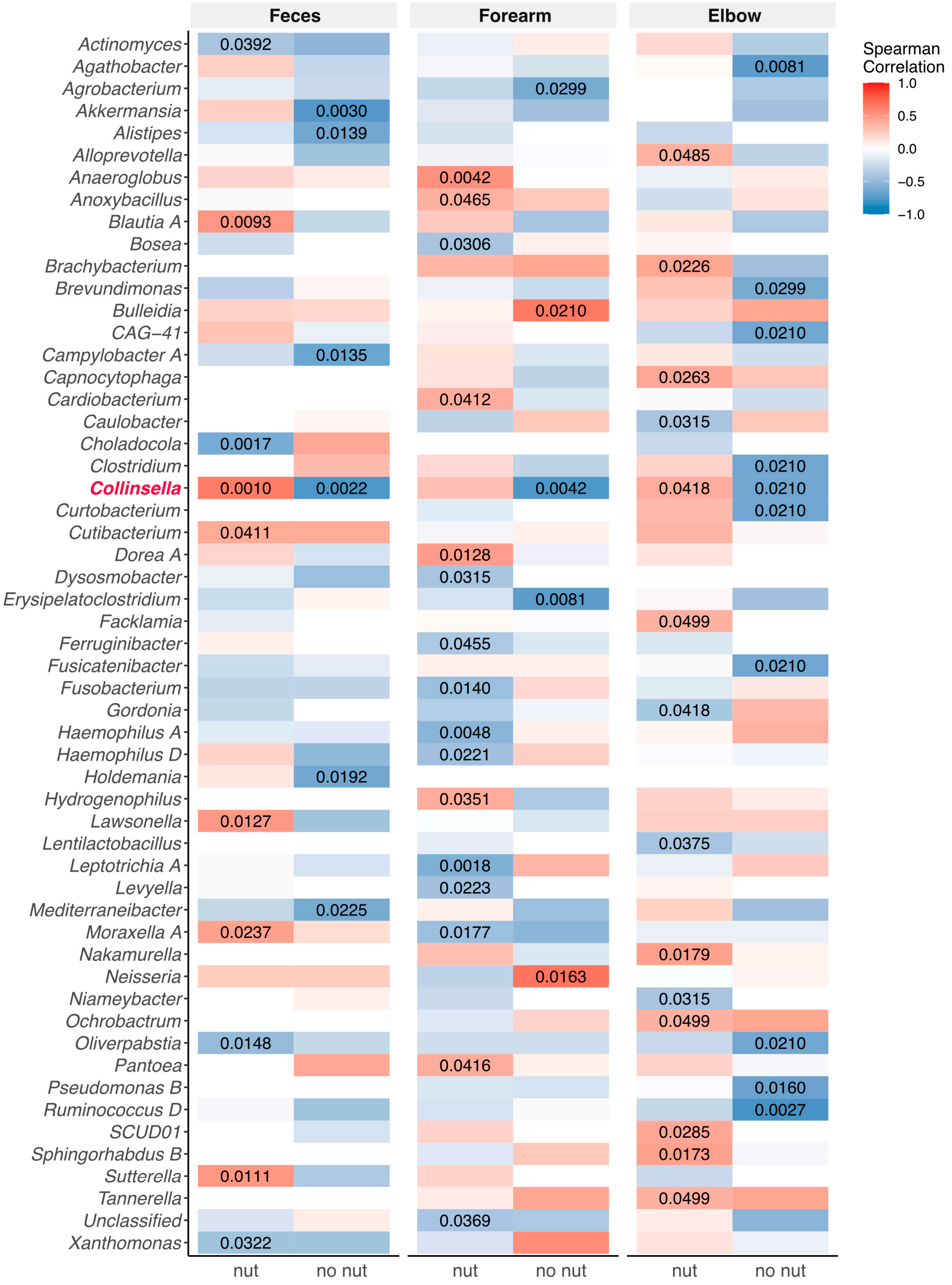

Supplementary Figure S6. Correlation of gut and skin microbiome of patients with tree nut and/or peanut allergy and total IgE. Spearman correlation of genera abundances and high total IgE, stratified according to the type of IgE-mediated food allergy in feces, forearm, and elbow samples (x-axis: nut, tree nut and/or peanut allergy, n = 15; no nut, an IgE-mediated food allergy other than tree nut and/or peanut allergy, n = 8; only significant p-values are shown ($p < 0.05$); red, positive correlation; blue, negative correlation, investigated time point t0).
